# Monitoring postoperative lung recovery using electrical impedance tomography in post anesthesia care unit: an observational study

**DOI:** 10.1007/s10877-021-00754-5

**Published:** 2021-09-20

**Authors:** Nadine Hochhausen, Torsten Kapell, Martin Dürbaum, Andreas Follmann, Rolf Rossaint, Michael Czaplik

**Affiliations:** Department of Anesthesiology, Medical Faculty, RWTH Aachen University, Aachen, Germany

**Keywords:** Electrical impedance tomography, Lung, Pulmonary atelectasis, Recovery room

## Abstract

With electrical impedance tomography (EIT) recruitment and de-recruitment phenomena can be quantified and monitored at bedside. The aim was to examine the feasibility of EIT with respect to monitor atelectasis formation and resolution in the post anesthesia care unit (PACU). In this observational study, 107 postoperative patients were investigated regarding the presence and recovery of atelectasis described by the EIT-derived parameters Global Inhomogeneity Index (GI Index), tidal impedance variation (TIV), and the changes in end-expiratory lung impedance (ΔEELI). We examined whether the presence of obesity (ADP group) has an influence on pulmonary recovery compared to normal weight patients (NWP group). During the stay at PACU, measurements were taken every 15 min. GI Index, TIV, and ΔEELI were calculated for each time point. 107 patients were monitored and EIT-data of 16 patients were excluded for various reasons. EIT-data of 91 patients were analyzed off-line. Their length of stay averaged 80 min (25th and 75th quartile 52–112). The ADP group demonstrated a significantly higher GI Index at PACU arrival (p < 0.001). This finding disappeared during their stay at the PACU. Additionally, the ADP group showed a significant increase in ΔEELI between PACU arrival and discharge (p = 0.025). Furthermore, TIV showed a significantly lower value during the first 90 min of PACU stay as compared to the time period thereafter (p = 0.036). Our findings demonstrate that obesity has an influence on intraoperative atelectasis formation and de-recruitment during PACU stay. The application of EIT in spontaneously breathing PACU patients seems meaningful in monitoring pulmonary recovery.

## Introduction

The risks for postoperative pulmonary complications are multifactorial, including patient-related factors like obesity [1]. A more frequent postoperative pulmonary complication is atelectasis formation that occur usually in the dependent parts of the lungs of most anesthetized patients [2, 3]. Especially in obese and severely obese patients, the incidence and the extend of atelectasis formation is increased [4]. In addition to the use of high oxygen concentration and the loss of muscle tone, surfactant depletion and the compression of lung tissue facilitate atelectasis formation and impair postoperative lung recovery [5]. Currently, postoperative pulmonary recovery is estimated by the course of respiratory rate (RR) and peripheral oxygen saturation (SpO_2_). But, pulmonary function cannot be represented by only these parameters. An increased difficulty in breathing, a pathological breathing pattern or a ventilation distribution disorder may lead to pulmonary complications and, in worst case scenarios, to reintubation and mechanical ventilation [6]. At the moment, only computer tomography (CT) is able to diagnose atelectasis and recruitment/de-recruitment phenomena reliably [7]. But, certainly, it is unfeasible to perform CT scans periodically in order to monitor recovery from atelectasis formation intra- and immediately postoperative. Radiation exposure and high resource requirements rule this method out as well.

Electrical impedance tomography (EIT) is a radiation-free, bedside-available, and non-invasive technique, which is able to visualize ventilation in time and spatial domains [8] and has already been studied in many areas related to ventilation [9, 10]. After reconstruction of the EIT images, impedance variations are visualized enabling monitoring of aeration and tidal ventilation [11]. It is already known that EIT can monitor atelectasis caused by a ventral-ward shift of impedance variation [12]. However, EIT-directed monitoring and appropriate therapy of the lungs have not yet entered into a clinical routine. Three of the frequently published parameters are the Global Inhomogeneity Index (GI Index) [13], which characterizes the inhomogeneity of the lung, the tidal impedance variation (TIV) [14], and the changes in end-expiratory lung impedance (ΔEELI) [15], which may be associated with recruitment or de-recruitment phenomena. Our hypothesis is that these EIT-derived parameters are able to visualize recovery from intra- and postoperative atelectasis formation.

The aim of this prospective observational study was to evaluate the feasibility of EIT in postoperative, spontaneously breathing obese and normal weight patients and to examine the presence and resolution of atelectatic lung areas by different EIT-derived indices, namely the GI index, TIV and ΔEELI.

## Materials and methods

### Study design and population

This observational study was approved by the local ethics committee (EK 058/13) as a clinical application study and took place in the PACU at the University Hospital Aachen.

From April to July 2013, 107 postoperative patients were informed about the study. Immediately after arriving at PACU and giving informed consent for the participation, data collection started. The patients were randomly chosen by allocation of the postoperative monitored beds equipped with the EIT. The inclusion criteria were ages 18 and above and the ability to give consent. In contrast, the exclusion criteria were ages below 18, pregnancy, implanted pacemakers or cardioverter-defibrillators, thoracic surgery in which there was a large surface wound, and a body mass index (BMI) above 50.

### Measurement, data acquisition, and study protocol

After admission to the PACU, different standardized data sheets were used including information about sex, height, weight, BMI, and time in surgery among other things as well as vital information and a checklist for EIT measurements. Subsequently, this data sheet was collected every 15 min for the duration of the PACU stay. To record continuous vital data (ECG, noninvasive blood pressure, oxygen saturation, respiratory rate), patients were connected to a Philips IntelliVue MP30 monitor (Philips Electronics N.V., Amsterdam, Netherlands). A 16-electrode EIT belt was fastened around the thorax and connected to the 5th intercostal space, which was attached to the PulmoVista500 (Draeger Medical, Luebeck, Germany). An EIT sequence of 2 min was recorded at every time point. Data Review 5.0 software (Draeger Medical, Luebeck, Germany) was utilized for acquisition and image reconstruction of the so-called tidal variation images, which represent the impedance difference between end-inspiration and end-expiration.

Prior to the discharge from the PACU, which followed internal hospital standards, final data acquisition was performed.

### Offline EIT analysis

The GI Index, TIV, and ΔEELI were calculated off-line using the EIT Diag v1.6 (Draeger Medical, Luebeck, Germany), according to Zhao et al. [13] and Bikker et al. [15].

The GI Index represents the heterogeneity of the lung. The ideal value would be 0, displaying a completely homogeneous ventilation. Disorders in ventilation distribution are reflected in higher GI Indices [13]. TIV, which correlates with gas volume changes in the lung [14], was calculated by selecting 10 subsequent breathing cycles from the recorded EIT sequences. Thereafter, differences in global impedance between end-inspiration and end-expiration were calculated, then averaged, and set in relation to the baseline measurement. Initial baseline measurement was defined as 100%. In conjunction with TIV, ΔEELI, which may be associated with recruitment/de- recruitment phenomena [15], was calculated and referred to the baseline measurement using the same preselected breathing cycles as used in TIV calculations.

### Statistical analysis

No power analysis was performed due to the observational nature of this study. First, the GI Index, TIV, and ΔEELI were analyzed and investigated in all patients. Second, patients were assigned to the obesity group (ADP), defined as having a BMI of more than 30, or to the normal weight patient group (NWP) with a BMI less than 30. Then, differences between arrival and discharge from the PACU, as well as the course of these EIT derived parameters during the stay, were considered. The groups were defined to indicate differences in atelectasis formation and their recovery due to preexisting obesity in contrast to normal weight. In addition, the time points (measurements in the recovery room were performed every 15 min) were clustered into two time periods (0 to 75 min, and 90 to 180 min) for comparing TIV trends. The Wilcoxon Test was used for repetitive parameter assessment (PACU arrival and discharge). The Mann–Whitney-*U*-Test was used to analyze differences between the two independent groups (ADP vs. NWP).

All data were analyzed with SPSS Statistics 23 for Windows (SPSS Inc., IBM Business Analytics Software, Armonk, NY, USA). All tests were two-tailed. Statistical significance was considered for p < 0.05. A Kolmogorov-–Smirnov test was used to confirm the not-normal distribution of the data. Therefore, median and interquartile ranges were calculated. The graphics were created using GraphPad Prism. Version 8.00 (GraphPad Software, La Jolla California USA).

## Results

Initially, 107 patients were included in the study, with their data recorded in the PACU and analyzed afterwards. One patient declined further data acquisition after the initial measurement because of feeling constricted by the EIT belt. All other patients remained in the study until their discharge from PACU. In total, sixteen patients were excluded post hoc for various reasons, such as preferred lateral position in the recovery room (three patients), unobtainable baseline measurements due to agitation (two patients), limited skin contact of the belt by bandage (one patient) or patient related factors like pre-existing comorbidities such as COPD and asthma, which require partly intermittent therapy in the recovery room (ten patients).

The analyzed study population, consisting of 33 female and 58 male subjects, exhibited a median body mass index (BMI) of 26.4 (25th and 75th quartile 22.2–30.4). Further demographic data are shown in Table 1.

**Table 1:** Demographic and clinical data

	ADP	NWP
Height (cm)	175 (163–183)	174 (164–180)
Weight (kg)	104 (95–117)	70 (60–80)
Body mass index (BMI)	32.8 (31.2–37.3)	23.7 (21.3–26.6)
Stay in the PACU (min)	80 (55–105)	79 (50–115)
Type of surgery		
Trauma surgery	2	4
Orthopaedics	3	8
Ear, nose and throat surgery	9	14
Oral and maxillofacial surgery	2	6
Vascular surgery	0	3
Plastic surgery	0	2
Operative gynaecology	1	4
Neurosurgery	0	4
General surgery	3	11
Ophthalmology	3	2
Urology	4	6

Data are stated as median and the 25th and 75th quartileor numbers

During their stay in the PACU for a median period of 80 min (25th and 75th quartile 52–112), vital parameters did not change significantly. Of these study population, twenty-seven patients were defined as obese (ADP group), sixty-four patients were normal in weight (NWP group), according to the predefined criteria. The BMI in ADP group was 32.8 (25th and 75th quartile 31.2–37.3) and in NWP group 23.7 (25th and 75th quartile 21.3–26.6). The PACU stay did not differ between the groups (ADP group: 80 (25th and 75th quartile 55–105) min and NWP group: 79 (25th and 75th quartile 50–115) min; p = 0.930).

**Fig. 1:**
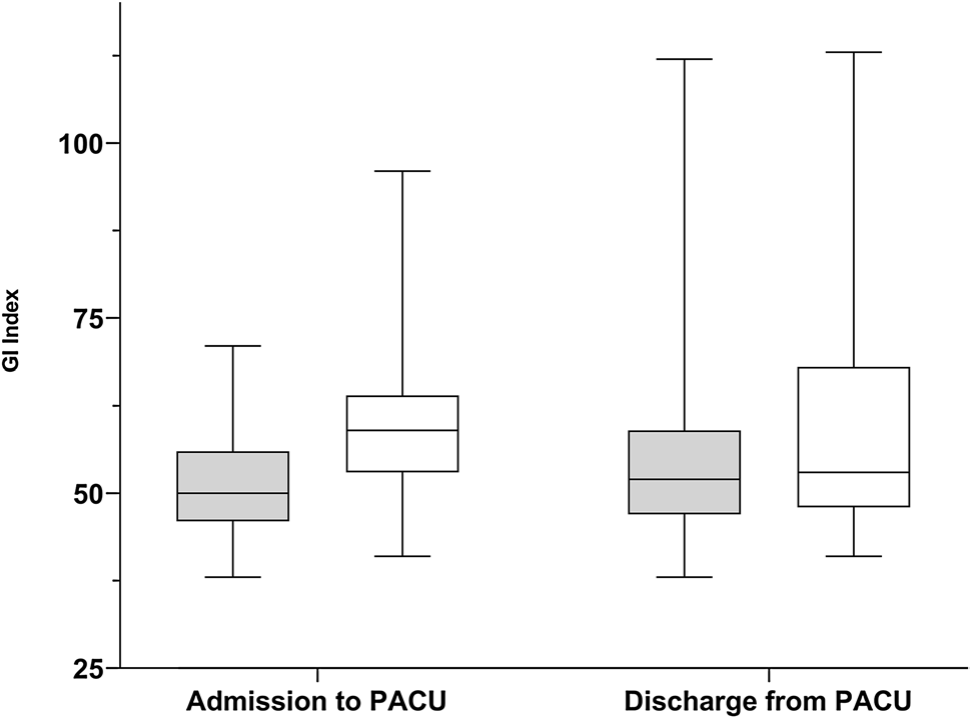
Global Inhomogeneity Index (GI Index) at arrival to and discharge from the post anesthesia care unit (PACU). The box plot shows the GI Index at admission and discharge from the PACU. At admission, there is a significant difference between obese (ADP group; white) and normal weight patients (NWP group; grey). At discharge, this difference is no longer visible

At PACU arrival, the following results were obtained: No statistical differences in vital parameters were shown between the ADP and the NWP group (Table 2), whereas the GI Index of the ADP group was higher as compared to the NWP group (59 (25th and 75th quartile 53–64) vs. 50 (25th and 75th quartile 46–56); p < 0.001) (Fig. 1).

**Table 2:** Vital parameter and EIT-indices of the obesity group and the normal weight group

	Admission to PACU			Discharge from PACU		
	ADP	NWP	p-value	ADP	NWP	p-value
Blood pressure systolic (mmHg)	124 (112–140)	133 (118–152)	0.206	124 (117–140)	133 (113–147)	0.603
Heart rate (bpm)	69 (61–82)	75 (63–80)	0.722	68 (60–77)	72 (62–80)	0.514
Respiratory rate (/min)	14 (12–19)	15 (12–17)	0.765	16 (13–18)	14 (13–17)	0.124
Oxygen saturation (%)	97 (93–99)	97 (93–99)	0.913	96 (94–98)	97 (94–98)	0.540
GI	59 (53–64)	50 (46–56)	**< 0.001**	53 (48–62)	52 (47–59)	0.602
ΔEELI (%)				34 (− 28 to 121)	15 (− 34 to 60)	0.185
TIV (%)				118 (84–181)	93 (73–121)	0.084

A statistical significance is highlighted in bold

Data are stated as median and the 25th and 75th quartile

Vital parameter and changes of end- expiratory lung impedance (ΔEELI), Global Inhomogeneity Index (GI Index) and tidal impedance variation (TIV) are given for the time point “admission” to and “discharge” from post anesthesia care unit (PACU) for obesity group (ADP) and normal weight group (NWP). GI Index differed significantly between ADP and NWP at PACU arrival. This effect disappeared during PACU stay. Data are stated as median values and interquartile ranges. Mann–Whitney-*U*-Test was used to analyze differences between two independent groups (ADP vs. NWP)

**Fig. 2:**
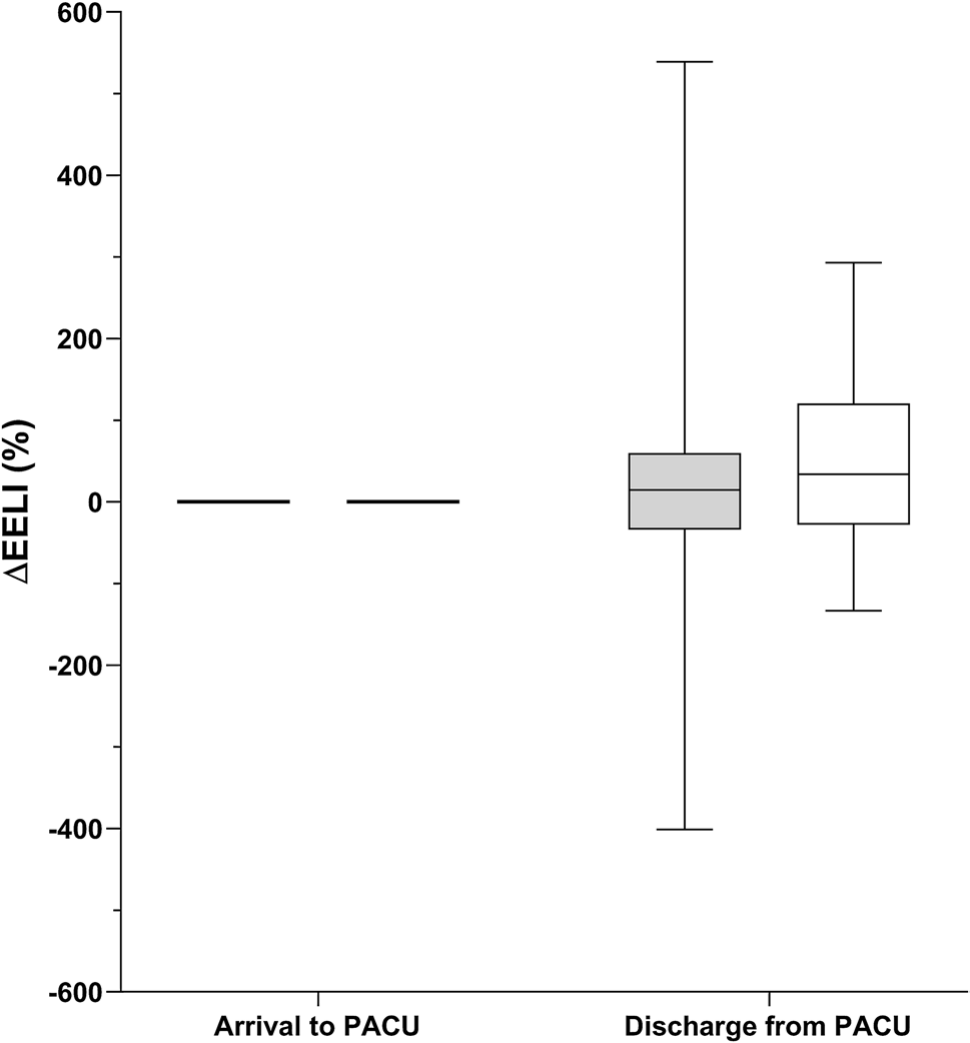
End-expiratory lung impedance (ΔEELI) at discharge from the post anesthesia care unit (PACU). The box plot shows the ΔEELI at discharge from the PACU. During PACU stay, the obese patients (ADP group; white) showed a significant increase in ΔEELI between PACU arrival and discharge. In normal weight patients (NWP group; grey), no significant change occurred.

**Fig. 3:**
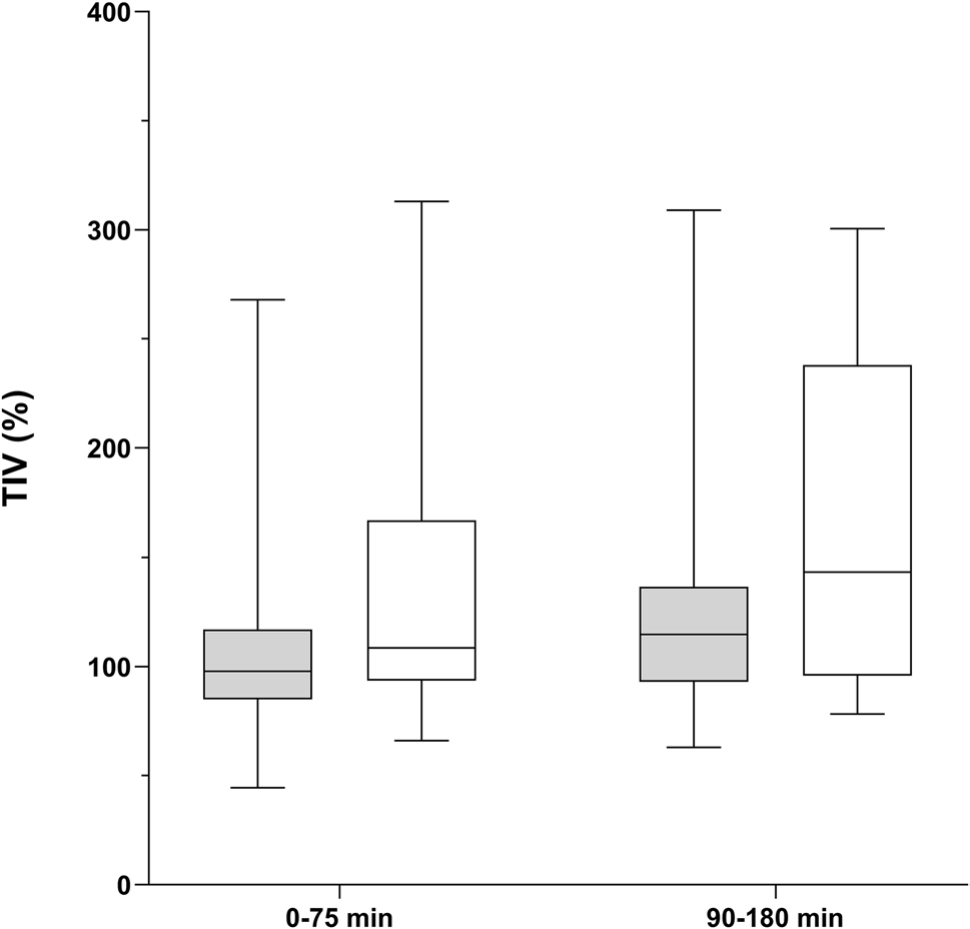
Tidal impedance variation (TIV) at different time periods.The box plot shows the statistical difference of the TIV comparing the first time period (0–75 min) to the second time period (90–180 min) in obese patients (ADP group; white). In normal weight patients (NWP group; grey), no significant change occurred

During PACU stay, the ADP group showed a significant increase in ΔEELI between PACU arrival and discharge, illustrating a recruitment (34% (25th and 75th quartile − 28 to 121) p = 0.025). In NWP group, no significant change occurred (15% (25th and 75th quartile − 34 to 60) p = 0.328) (Fig. 2). Additionally, the TIV did not differ significantly between PACU arrival and discharge, neither in ADP (p = 0.064) nor in NWP group (p = 0.343). But, the TIV showed a statistical increase in the second time period (90 to 180 min) when compared to the first time period (0 to 75 min) in the ADP group from 109 (25th and 75th quartile 94–167) to 143 (25th and 75th quartile 96–238) p = 0.036). This effect did not occur in the NWP group (from 98 (25th and 75th quartile 85–122) to 115 (25th and 75th quartile 93–158) p = 0.124) (Fig. 3). However, the GI Index showed a significant increase from 50 (25th and 75th quartile 46–56) to 52 (25th and 75th quartile 47–59); p = 0.008) in NWP group during PACU stay, whereas in the ADP group, no statistical difference was demonstrated (p = 0.247).

At PACU discharge, GI index did not demonstrate anymore a statistically significance between the groups (GI Index: 53 (25th and 75th quartile 48–62) in the ADP group vs. 52 (25th and 75th quartile 47–59) in the NWP group; p = 0.602). Additionally, vitals, ΔEELI (p = 0.185), and TIV (p = 0.084) did not differ as well (Table 2; Fig. 1).

## Discussion

In this clinical study, obese and NWPs were compared in terms of postoperative recovery. Ventilation was visualized and analyzed with EIT; therewith, measures and parameters representing atelectasis and ventilation impairment were obtained. Obesity was found out to have a significant influence on GI Index at PACU arrival. During PACU stay, the inhomogeneity decreased significantly over time in the ADP group. This might be related with recruitment effects.

The following three major findings of the current study should be discussed: (1) ΔEELI significantly increased during the PACU stay in ADP; (2) when comparing the time periods of (A) the first 90 min (median) and (B) 90 to 180 min, TIV was higher during (B) in the ADP group as well; (3) initially, GI differed between NWP and ADP, but not anymore after discharge from PACU.

One reason for the increasing ΔEELI over time might be related to atelectasis formation. Probably, this could be due to a remaining influence of intraoperative anesthetics leading to a lower respiratory drive [16–18]. The hypothesis about a remaining effect of anesthetics is also supported by the course of TIV. After clustering the time points in two time periods ((A) the first 90 min (median) and (B) 90 to 180 min), it became obvious that in the second time period the TIV was significantly higher compared to the TIV in the first time period in the ADP group. One possible explanation might be the fact that just after arrival at the PACU, tidal volume was low and the patient’s breathing was shallow due to the lingering effects of opioids, anesthetics, and muscle relaxants. For that reason, TIV is low according to a low tidal volume. After complete drug metabolising and recovered vigilance, the TIV increased with consecutive recruitment effects. This may result in a more homogeneous ventilation, characterized by a lower GI Index in the ADP group patients at discharge compared to their arrival. In order to counteract these changes, the OXYBAR study examines whether a postoperative high-flow nasal oxygen therapy compared to conventional oxygen therapy in obese patients can improve respiratory function, determined by an increase of ΔEELI [19]. Unfortunately, we do not have individualized reference measurements before anesthesia or information about intraoperative ventilation. However, a significant increase of the GI Index was observed in the NWP group between PACU arrival and discharge. One possible explanation for this is that more attention is paid to obese patients in the PACU, as they are known to be at risk of respiratory problems postoperatively. For this reason, care is taken to ensure that the upper body is adequately elevated. In addition, this group of patients is motivated to perform deep inspiration and expiration. Normal-weight patients, on the other hand, are not as concerned about postoperative respiratory problems. Probably, less attention is paid to optimal positioning. In addition, deep breathing is not as frequently remembered. This could be the cause of the slight increase in the GI Index in the NWP group between PACU arrival and discharge which is most likely not clinically relevant.

Generally, all patients showed a ventilation impairment in terms of inhomogeneous ventilation due to atelectasis formation after anesthesia induction [2]. The intraoperative supine position, in combination with mechanical ventilation, may have been responsible for the presence of atelectasis. Typically, ventilation moves ventral-wards in this situation [20, 21]. It is known that obese patients tend to atelectasis formation even before anesthesia induction [3, 20]. The appropriate positive end-expiratory pressure (PEEP) application might prevent intraoperative atelectasis, but not after extubation [22]. Here, obese patients may benefit from an intraoperative individualized PEEP [23]. Furthermore, a randomized clinical trial shows that the choice of an intraoperative high PEEP level combined with alveolar recruitment maneuvers is not associated with a reduction of postoperative pulmonary complications compared to an intraoperative lower PEEP level in obese patients [24]. At their PACU arrival, shortly after extubation, the ADP group patients demonstrated a more heterogeneous ventilation prefiguring atelectasis formation, which was represented by a higher GI Index. Additionally, at PACU discharge, the ΔEELI tended to be higher in the ADP group. So, the potential ongoing postoperative effect of the application of an intraoperative PEEP remains unclear, since this was not examined in this study. But it is a fact that after extubation, the PEEP effect vanishes immediately [25]. Additionally, obese patients, especially those suffering from obstructive sleep apnea, tend to have upper airway obstruction after pain relief [26] because of the prolonged elimination half-life of sufentanyl or fentanyl [27]. In addition, correct dosage for obese patients is challenging [28]. The common path is an impaired respiration with atelectasis formation as it was demonstrated in our study.

There are several limitations in this study that must be addressed. First, using only a few exclusion criteria, a heterogeneous study population resulted in our study. But, Marquis et al. showed that body characteristics like height, weight, BMI, and thoracic parameters do not seem to have a technical influence on EIT measurements [29]. Second, the time periods employed in the PACU varied extensively between individual patients as a result of surgeries and previous illnesses. We, therefore, compared arrival and discharge in each case specifically and not at a fixed time point. Third, only one EIT-belt was used, which was positioned in the 5th intercostal space. The 5th intercostal space seems to be one of the best electrode planes for estimating global lung parameters [30]. In addition, several studies indicate that a measurement with an EIT-belt in the 5th intercostal space reflects the heterogeneity of the whole lung in the ventro-dorsal direction [31]. Additionally, posture may affect EIT measurements [32]. In our study, all patients were rested with their upper body elevated. So, EIT measurements were comparable inter-individually. Fourth, EIT indices were developed for mechanically ventilated and sedated patients. In contrast, patients in our study were awake and breathing spontaneously, perhaps with noticeable breathing patterns relating to stress, pain, analgesics as well as the lingering effects of anesthetics. Therefore, appropriate sequences of at least 10 breathing cycles were selected manually for signal analysis. Additionally, the patients were sitting with their upper body elevated (due to clinical standards in the PACU), close to an upright sitting position, which seems to be the preferred position for EIT patients when awake [33]. However, even different ventilation modes can affect EIT measurements [34–36]. Finally, no individual reference, ideally before the induction of anesthesia, was recorded. Further clinical studies of this type should include a preoperative baseline measurement and intraoperative reference data. In addition, the knowledge of intraoperative ventilation parameters as well as the implementation of intraoperative recruitment maneuvers may be helpful for interpreting postoperative EIT measurements.

## Conclusions

To sum up, our data demonstrate the feasible use of EIT in postoperative patients in the PACU. After a long time period of animal trials demonstrating the general potentials of EIT, nowadays the technique is also used for PEEP guidance in various experimental and clinical studies [37–39]. Recently, EIT was used to predict failure of spontaneous breathing trials [40]. Furthermore, EIT visualized that a sitting position and exercise increase lung aeration [41]. Equally, EIT illustrates an increased functional residual capacity in noninvasive ventilation during spontaneous breathing anesthesia [42]. Therefore, the application of EIT might be useful to monitor postoperative recovery in terms of adequate respiration. In future, a combination of different EIT- derived indices may be useful to find out the best time point for discharging the patients from the PACU, especially in obese patients. Our data show that obese patients tend to have a longer phase of de-recruitment and inhomogeneous ventilation. Beside the GI Index, the ΔEELI and the TIV, the intra-tidal gas distribution [31] seems to be a promising index. So, EIT may help to figure out the time point when the de-recruitment phase ends, gas volume in the lung increases and ventilation gets more homogeneous.
